# Supplementary material for: Integrated Transcriptomics and Metabolomics Reveal the Mechanism of Alliin in Improving Hyperlipidemia
Source: Foods. 2023 Sep 13;12(18):3407. doi: 10.3390/foods12183407 (PMC10528980; doi:10.3390/foods12183407)
Supplement: Supplementary file 1 [file foods-12-03407-s001.zip › foods-2598020-SI.pdf]

Integrated transcriptomics and metabolomics reveal the mechanism of alliin in improving hyperlipidemia

Min Zhang[ab], Xiaoying Zou[ab], Yixuan Du[ab], Zhuangguang Pan[ab], Fangqing He[ab], Yuanming Sun[ab], *, Meiying Li[ab], *

a Guangdong Provincial Key Lab of Food Safety and Quality, South China Agricultural University, Guangzhou, Guangdong 510642, China

b College of Food, South China Agricultural University, Guangzhou 510642, China

* Corresponding author. Meiying Li: E-mail:lmy1982@scau.edu.cn
Guangdong Provincial Key Lab of Food Safety and Quality, South China Agricultural University, China. Tel: +86 20 85283448 Fax: +86 20 85280270

Table S1 diet formulation in mice

diet number	GD450B		GD60	
name	35% sucrose control feed		60% high-fat feed	
Typical analysis	Mass ratio (%)	Energy ratio (%)	Mass ratio (%)	Energy ratio (%)
Protein	19.2	20	26	20
Carbohydrate	67.3	70	26	20
Fat	4.3	10	35	60
sum	/	100	/	100
Kcal/g	3.85	/	5.24	/
Product	Mass ratio (g/kg)	kcal	Mass ratio (g/kg)	Kcal
Casein	200	800	200	800
L-Cystine	3	12	3	12
Com Starch	315	1260	0	0
Maltodextrin	35	140	125	500
Sucrose	345	1380	63.8	255.2
Cellulose	50	0	50	0
Soybean Oil	25	225	25	225
Lard	20	180	245	2205
Mineral Mix S10026B	50	36	50	36

Vitamin Mix AIN-76A	10	39	10	39
Choline Bitartrate	2	0	2	0
sum	1055	4072	738.8	4072.2

Table S2 PCR primer sequences of gene related to liver lipid metabolism

Gene name	Forward	Reverse
Sqle	AGTTCGCTGCCTTCTCGGATA	GCTCCTGTTAATGTCGTTTCTGA
Cel	TGGAGGGCGTCAACAAGAAG	GTGGCCTGTAGGCATCGTTT
Ugt1a2	ATGGACACGGGACTATGTGTG	CATGGGTAACACCAGCACTTTT
Myc	ATGCCCCTCAACGTGAACTTC	CGCAACATAGGATGGAGAGCA
β-actin	GTGACGTTGACATCCGTAAAGA	GCCGGACTCATCGTACTCC

Table S3 Transcriptome analysis of significant difference genes among the three group, according to the criteria of FC>=2 and p<0.05

Gene id	Gene name	Gene description	HFD_vs_control	alliin_vs_HFD	Sum
Genes Number	/	/	405	148	514
ENSMUS	Tmem2	transmembrane protein 215	yes|down	no|up	1

G00000046593	15	[Source:MGI Symbol;Acc:MGI:2444167]			
ENSMUSG00000112824	Gm47917	predicted gene, 47917 [Source:MGI Symbol;Acc:MGI:6097168]	yes|down	no|up	1
ENSMUSG00000074115	Saa1	serum amyloid A 1 [Source:MGI Symbol;Acc:MGI:98221]	no|up	yes|down	1
ENSMUSG00000064358	mt-Co3	mitochondrially encoded cytochrome c oxidase III [Source:MGI Symbol;Acc:MGI:102502]	yes|down	yes|up	2
ENSMUSG00000038893	Fam117a	family with sequence similarity 117, member A [Source:MGI Symbol;Acc:MGI:2144564]	yes|down	no|up	1
ENSMUSG00000087020	Gm16283	predicted gene 16283 [Source:MGI Symbol;Acc:MGI:3826591]	no|down	yes|up	1
ENSMUSG00000027384	Ndufaf5	NADH:ubiquinone oxidoreductase complex assembly factor 5 [Source:MGI Symbol;Acc:MGI:1916737]	yes|down	no|up	1

ENSMUSG00000046691	Chtf8	CTF8, chromosome transmission fidelity factor 8 [Source:MGI Symbol;Acc:MGI:2443370]	no|up	yes|down	1
ENSMUSG00000056978	Hamp2	hepcidin antimicrobial peptide 2 [Source:MGI Symbol;Acc:MGI:2153530]	yes|down	no|up	1
ENSMUSG00000038393	Txnip	thioredoxin interacting protein [Source:MGI Symbol;Acc:MGI:1889549]	yes|up	no|down	1
ENSMUSG00000026249	Serpine2	serine (or cysteine) peptidase inhibitor, clade E, member 2 [Source:MGI Symbol;Acc:MGI:101780]	yes|up	no|down	1
ENSMUSG00000114710	Gm40923	predicted gene, 40923 [Source:MGI Symbol;Acc:MGI:5623808]	yes|up	no|down	1
ENSMUSG00000109887	Gm28756	predicted gene 28756 [Source:MGI Symbol;Acc:MGI:5579462]	no|down	yes|up	1
ENSMUSG00000031517	Gpm6a	glycoprotein m6a [Source:MGI Symbol;Acc:MGI:107671]	yes|down	yes|up	2

ENSMUSG00000050860	Phospho1	phosphatase, orphan 1 [Source:MGI Symbol;Acc:MGI:2447348]	yes|up	no|down	1
ENSMUSG00000017146	Brca1	breast cancer 1, early onset [Source:MGI Symbol;Acc:MGI:104537]	no|down	yes|down	1
ENSMUSG00000089929	Bcl2a1b	B cell leukemia/lymphoma 2 related protein A1b [Source:MGI Symbol;Acc:MGI:1278326]	yes|down	no|down	1
ENSMUSG00000076564	Igkv12-46	immunoglobulin kappa variable 12-46 [Source:MGI Symbol;Acc:MGI:4439773]	yes|down	no|down	1
ENSMUSG00000031861	Lpar2	lysophosphatidic acid receptor 2 [Source:MGI Symbol;Acc:MGI:1858422]	yes|down	no|up	1
ENSMUSG00000015127	Unkl	unkempt family like zinc finger [Source:MGI Symbol;Acc:MGI:1921404]	yes|up	no|down	1
ENSMUSG00000097730	Gm26588	predicted gene, 26588 [Source:MGI Symbol;Acc:MGI:5477082]	yes|up	no|down	1
ENSMUS	Gm503	predicted gene, 50388	no|up	yes|down	1

G00000117789	88	[Source:MGI Symbol;Acc:MGI:6303291]			
ENSMUSG00000000876	Pxmp4	peroxisomal membrane protein 4 [Source:MGI Symbol;Acc:MGI:1891701]	yes|up	no|down	1
ENSMUSG00000104806	Gm42566	predicted gene 42566 [Source:MGI Symbol;Acc:MGI:5662703]	no|down	yes|up	1
ENSMUSG00000032098	Treh	trehalase (brush-border membrane glycoprotein) [Source:MGI Symbol;Acc:MGI:1926230]	yes|up	no|down	1
ENSMUSG00000006445	Epha2	Eph receptor A2 [Source:MGI Symbol;Acc:MGI:95278]	yes|up	no|down	1
ENSMUSG00000087658	Hotairm1	Hoxa transcript antisense RNA, myeloid-specific 1 [Source:MGI Symbol;Acc:MGI:3705155]	yes|down	no|up	1
ENSMUSG00000035042	Ccl5	chemokine (C-C motif) ligand 5 [Source:MGI Symbol;Acc:MGI:98262]	yes|down	no|up	1
ENSMUS	Ackr2	atypical chemokine receptor 2	yes|up	no|down	1

G00000044534		[Source:MGI Symbol;Acc:MGI:1891697]			
ENSMUSG00000017734	Dbndd2	dysbindin (dystrobrevin binding protein 1) domain containing 2 [Source:MGI Symbol;Acc:MGI:106562]	yes|down	no|up	1
ENSMUSG00000003477	Inmt	indolethylamine N-methyltransferase [Source:MGI Symbol;Acc:MGI:102963]	yes|down	no|up	1
ENSMUSG00000068129	Cst7	cystatin F (leukocystatin) [Source:MGI Symbol;Acc:MGI:1298217]	no|down	yes|down	1
ENSMUSG00000022003	Slc25a30	solute carrier family 25, member 30 [Source:MGI Symbol;Acc:MGI:1914804]	yes|up	no|up	1
ENSMUSG00000110899	Gm48840	predicted gene, 48840 [Source:MGI Symbol;Acc:MGI:6098571]	no|down	yes|up	1
ENSMUSG00000021765	Fst	follistatin [Source:MGI Symbol;Acc:MGI:95586]	yes|down	no|up	1

ENSMUSG00000042834	Nrep	neuronal regeneration related protein [Source:MGI Symbol;Acc:MGI:99444]	yes|down	no|up	1
ENSMUSG00000084839	Gm14097	predicted gene 14097 [Source:MGI Symbol;Acc:MGI:3651729]	yes|up	no|down	1
ENSMUSG00000018199	Ro60	Ro60, Y RNA binding protein [Source:MGI Symbol;Acc:MGI:106652]	no|down	yes|up	1
ENSMUSG00000020538	Srebf1	sterol regulatory element binding transcription factor 1 [Source:MGI Symbol;Acc:MGI:107606]	yes|down	no|down	1
ENSMUSG00000108884	Gm45792	predicted gene 45792 [Source:MGI Symbol;Acc:MGI:5804907]	yes|down	no|up	1
ENSMUSG00000113386	Gm47357	predicted gene, 47357 [Source:MGI Symbol;Acc:MGI:6096262]	no|down	yes|up	1
ENSMUSG00000044748	Defb1	defensin beta 1 [Source:MGI Symbol;Acc:MGI:1096878]	no|down	yes|up	1

ENSMUSG00000078866	Zfp970	zinc finger protein 970 [Source:MGI Symbol;Acc:MGI:3652255]	yes|down	no|up	1
ENSMUSG00000045211	Nudt18	nudix (nucleoside diphosphate linked moiety X)-type motif 18 [Source:MGI Symbol;Acc:MGI:2385853]	yes|up	no|down	1
ENSMUSG00000032561	Acpp	acid phosphatase, prostate [Source:MGI Symbol;Acc:MGI:1928480]	yes|down	no|down	1
ENSMUSG00000070360	Amy2a1	amylase 2a1 [Source:MGI Symbol;Acc:MGI:104548]	yes|up	yes|down	2
ENSMUSG00000113475	Gm49366	predicted gene, 49366 [Source:MGI Symbol;Acc:MGI:6121579]	yes|up	yes|down	2
ENSMUSG00000113476	Gm48309	predicted gene, 48309 [Source:MGI Symbol;Acc:MGI:6097758]	no|down	yes|up	1
ENSMUSG00000092021	Gbp11	guanylate binding protein 11 [Source:MGI Symbol;Acc:MGI:3646307]	yes|down	yes|up	2

ENSMUSG00000094257	Ap3s1-ps2	adaptor-related protein complex 3, sigma 1 subunit, pseudogene 2 [Source:MGI Symbol;Acc:MGI:1929217]	yes|down	no|up	1
ENSMUSG00000048856	Slc25a47	solute carrier family 25, member 47 [Source:MGI Symbol;Acc:MGI:2144766]	yes|up	no|down	1
ENSMUSG00000032802	Srxn1	sulfiredoxin 1 homolog (S. cerevisiae) [Source:MGI Symbol;Acc:MGI:104971]	yes|up	no|down	1
ENSMUSG00000032806	Slc10a3	solute carrier family 10 (sodium/bile acid cotransporter family), member 3 [Source:MGI Symbol;Acc:MGI:95048]	no|up	yes|down	1
ENSMUSG00000038576	Susd4	sushi domain containing 4 [Source:MGI Symbol;Acc:MGI:2138351]	yes|up	no|down	1
ENSMUSG00000028655	Mfsd2a	major facilitator superfamily domain containing 2A [Source:MGI Symbol;Acc:MGI:1923824]	yes|up	yes|down	2
ENSMUS	Gpat3	glycerol-3-phosphate	yes|up	no|down	1

G00000029314		acyltransferase 3 [Source:MGI Symbol;Acc:MGI:3603816]			
ENSMUSG00000090925	1810064F22Rik	RIKEN cDNA 1810064F22 gene [Source:MGI Symbol;Acc:MGI:1917112]	yes|down	no|up	1
ENSMUSG00000112012	Gm47025	predicted gene, 47025 [Source:MGI Symbol;Acc:MGI:6095715]	no|down	yes|up	1
ENSMUSG00000027533	Fabp5	fatty acid binding protein 5, epidermal [Source:MGI Symbol;Acc:MGI:101790]	yes|down	no|up	1
ENSMUSG00000038225	Primpol	primase and polymerase (DNA-directed) [Source:MGI Symbol;Acc:MGI:3603756]	yes|down	no|up	1
ENSMUSG00000063388	BC023105	cDNA sequence BC023105 [Source:MGI Symbol;Acc:MGI:2384767]	yes|down	no|up	1
ENSMUSG00000029417	Cxcl9	chemokine (C-X-C motif) ligand 9 [Source:MGI Symbol;Acc:MGI:1352449]	yes|down	no|up	1
ENSMUSG0000009	Gm29668	predicted gene 29668 [Source:MGI	no|down	yes|up	1

9835		Symbol;Acc:MGI:5580374]			
ENSMUSG00000110353	Gm33543	predicted gene, 33543 [Source:MGI Symbol;Acc:MGI:5592702]	yes|up	no|up	1
ENSMUSG00000086296	D030055H07Rik	RIKEN cDNA D030055H07 gene [Source:MGI Symbol;Acc:MGI:3605036]	yes|up	no|down	1
ENSMUSG00000043421	Hilpda	hypoxia inducible lipid droplet associated [Source:MGI Symbol;Acc:MGI:1916823]	yes|up	no|up	1
ENSMUSG00000043542	Zc2hc1a	zinc finger, C2HC-type containing 1A [Source:MGI Symbol;Acc:MGI:1914556]	yes|down	yes|up	2
ENSMUSG00000035863	Palm	paralemmin [Source:MGI Symbol;Acc:MGI:1261814]	yes|down	no|up	1
ENSMUSG00000101655	2310040G24Rik	RIKEN cDNA 2310040G24 gene [Source:MGI Symbol;Acc:MGI:1916897]	yes|down	no|up	1
ENSMUSG00000095180	Rhox5	reproductive homeobox 5 [Source:MGI Symbol;Acc:MGI:97538]	yes|down	no|down	1

ENSMUSG00000041237	Pklr	pyruvate kinase liver and red blood cell [Source:MGI Symbol;Acc:MGI:97604]	yes|down	no|down	1
ENSMUSG00000091542	Gm17167	predicted gene 17167 [Source:MGI Symbol;Acc:MGI:4937994]	no|down	yes|up	1
ENSMUSG00000038827	Abitram	actin binding transcription modulator [Source:MGI Symbol;Acc:MGI:2677850]	yes|up	no|down	1
ENSMUSG00000109564	Muc16	mucin 16 [Source:MGI Symbol;Acc:MGI:1920982]	yes|down	no|up	1
ENSMUSG00000080316	Spaca6	sperm acrosome associated 6 [Source:MGI Symbol;Acc:MGI:1922452]	yes|down	no|up	1
ENSMUSG00000076569	Igkv5-39	immunoglobulin kappa variable 5-39 [Source:MGI Symbol;Acc:MGI:2686255]	yes|up	no|down	1
ENSMUSG00000097171	Gm17644	predicted gene, 17644 [Source:MGI Symbol;Acc:MGI:4937278]	no|down	yes|up	1
ENSMUS	Gm428	predicted gene 42857	yes|down	no|up	1

G00000106924	57	[Source:MGI Symbol;Acc:MGI:5662994]			
ENSMUSG00000097073	9430037G07Rik	RIKEN cDNA 9430037G07 gene [Source:MGI Symbol;Acc:MGI:2444544]	yes|up	no|up	1
ENSMUSG00000033618	Map3k13	mitogen-activated protein kinase kinase kinase 13 [Source:MGI Symbol;Acc:MGI:2444243]	yes|up	no|down	1
ENSMUSG00000039620	Trmt9b	tRNA methyltransferase 9B [Source:MGI Symbol;Acc:MGI:2442328]	yes|up	no|down	1
ENSMUSG00000102976	Zc3h11a	zinc finger CCCH type containing 11A [Source:MGI Symbol;Acc:MGI:1917829]	no|down	yes|up	1
ENSMUSG00000110411	Gm45457	predicted gene 45457 [Source:MGI Symbol;Acc:MGI:5791293]	no|down	yes|up	1
ENSMUSG00000032578	Cish	cytokine inducible SH2-containing protein [Source:MGI Symbol;Acc:MGI:103159]	yes|down	no|up	1
ENSMUS	Fut11	fucosyltransferase 11	yes|down	yes|up	2

G00000039357		[Source:MGI Symbol;Acc:MGI:1920318]			
ENSMUSG00000018796	Acsl1	acyl-CoA synthetase long-chain family member 1 [Source:MGI Symbol;Acc:MGI:102797]	yes|up	no|down	1
ENSMUSG00000090171	Ugt1a2	UDP glucuronosyltransferase 1 family, polypeptide A2 [Source:MGI Symbol;Acc:MGI:3576049]	no|down	yes|up	1
ENSMUSG00000038917	3930402G23Rik	RIKEN cDNA 3930402G23 gene [Source:MGI Symbol;Acc:MGI:1921277]	yes|up	no|up	1
ENSMUSG00000105081	Gm43110	predicted gene 43110 [Source:MGI Symbol;Acc:MGI:5663247]	yes|up	no|up	1
ENSMUSG00000066513	Klk1b4	kallikrein 1-related pepidase b4 [Source:MGI Symbol;Acc:MGI:97320]	yes|down	no|up	1
ENSMUSG00000052316	Lrrc15	leucine rich repeat containing 15 [Source:MGI Symbol;Acc:MGI:1921738]	yes|up	no|down	1
ENSMUS	Gm457	predicted gene 45732	no|down	yes|up	1

G00000110704	32	[Source:MGI Symbol;Acc:MGI:5804847]			
ENSMUSG00000060560	Ces4a	carboxylesterase 4A [Source:MGI Symbol;Acc:MGI:2384581]	no|up	yes|up	1
ENSMUSG00000039519	Cyp7b1	cytochrome P450, family 7, subfamily b, polypeptide 1 [Source:MGI Symbol;Acc:MGI:104978]	yes|up	no|up	1
ENSMUSG00000048728	Zfp454	zinc finger protein 454 [Source:MGI Symbol;Acc:MGI:2679253]	yes|down	no|up	1
ENSMUSG00000018566	Slc2a4	solute carrier family 2 (facilitated glucose transporter), member 4 [Source:MGI Symbol;Acc:MGI:95758]	yes|down	no|up	1
ENSMUSG00000110838	Gm47789	predicted gene, 47789 [Source:MGI Symbol;Acc:MGI:6096957]	no|down	yes|up	1
ENSMUSG00000057465	Saa2	serum amyloid A 2 [Source:MGI Symbol;Acc:MGI:98222]	no|up	yes|down	1

ENSMUSG00000085651	Gm11695	predicted gene 11695 [Source:MGI Symbol;Acc:MGI:3649841]	yes|up	yes|down	2
ENSMUSG00000056290	Ms4a4b	membrane-spanning 4-domains, subfamily A, member 4B [Source:MGI Symbol;Acc:MGI:1913083]	yes|down	no|up	1
ENSMUSG00000060950	Trmt61a	tRNA methyltransferase 61A [Source:MGI Symbol;Acc:MGI:2443487]	yes|up	no|down	1
ENSMUSG00000105909	Gm43189	predicted gene 43189 [Source:MGI Symbol;Acc:MGI:5663326]	no|down	yes|up	1
ENSMUSG00000044367	Slc16a13	solute carrier family 16 (monocarboxylic acid transporters), member 13 [Source:MGI Symbol;Acc:MGI:1916559]	yes|up	no|down	1
ENSMUSG00000085156	Snhg15	small nucleolar RNA host gene 15 [Source:MGI Symbol;Acc:MGI:3650059]	no|down	yes|up	1
ENSMUS	Mr1	major histocompatibility	yes|down	no|down	1

G00000026471		complex, class I-related [Source:MGI Symbol;Acc:MGI:1195463]			
ENSMUSG00000085154	C130046K22Rik	RIKEN cDNA C130046K22 gene [Source:MGI Symbol;Acc:MGI:3026935]	yes|up	no|down	1
ENSMUSG00000015143	Actn1	actinin, alpha 1 [Source:MGI Symbol;Acc:MGI:2137706]	yes|down	no|up	1
ENSMUSG00000019863	Qrsl1	glutaminyl-tRNA synthase (glutamine-hydrolyzing)-like 1 [Source:MGI Symbol;Acc:MGI:1923813]	yes|up	no|down	1
ENSMUSG00000039533	Mmd2	monocyte to macrophage differentiation-associated 2 [Source:MGI Symbol;Acc:MGI:1922354]	yes|down	no|down	1
ENSMUSG00000066072	Cyp4a10	cytochrome P450, family 4, subfamily a, polypeptide 10 [Source:MGI Symbol;Acc:MGI:88611]	yes|up	no|down	1
ENSMUS	Chrna4	cholinergic receptor, nicotinic,	yes|down	no|down	1

G00000027577		alpha polypeptide 4 [Source:MGI Symbol;Acc:MGI:87888]			
ENSMUSG00000029188	Slc34a2	solute carrier family 34 (sodium phosphate), member 2 [Source:MGI Symbol;Acc:MGI:1342284]	yes|up	no|down	1
ENSMUSG00000009633	G0s2	G0/G1 switch gene 2 [Source:MGI Symbol;Acc:MGI:1316737]	yes|up	no|down	1
ENSMUSG00000039114	Nrn1	neuritin 1 [Source:MGI Symbol;Acc:MGI:1915654]	yes|down	no|up	1
ENSMUSG00000058331	Zfp85	zinc finger protein 85 [Source:MGI Symbol;Acc:MGI:107767]	no|down	yes|up	1
ENSMUSG00000089635	Gm16559	predicted gene 16559 [Source:MGI Symbol;Acc:MGI:4414979]	no|down	yes|up	1
ENSMUSG00000057766	Ankrd29	ankyrin repeat domain 29 [Source:MGI Symbol;Acc:MGI:2687055]	yes|down	no|up	1

ENSMUSG00000101537	Gm29571	predicted gene 29571 [Source:MGI Symbol;Acc:MGI:5580277]	yes|down	no|down	1
ENSMUSG00000107352	Gm43660	predicted gene 43660 [Source:MGI Symbol;Acc:MGI:5663797]	yes|down	no|up	1
ENSMUSG00000029009	Mthfr	methylenetetrahydrofolate reductase [Source:MGI Symbol;Acc:MGI:106639]	yes|up	no|down	1
ENSMUSG00000082424	Gm13292	predicted gene 13292 [Source:MGI Symbol;Acc:MGI:3649470]	yes|down	yes|up	2
ENSMUSG00000039886	Tmem120a	transmembrane protein 120A [Source:MGI Symbol;Acc:MGI:2686991]	yes|up	no|down	1
ENSMUSG00000078451	Ppil6	peptidylprolyl isomerase (cyclophilin)-like 6 [Source:MGI Symbol;Acc:MGI:1920325]	no|down	yes|up	1
ENSMUSG00000020319	Wdpcp	WD repeat containing planar cell polarity effector [Source:MGI Symbol;Acc:MGI:2144467]	yes|down	no|up	1
ENSMUS	Dio3os	deiodinase, iodothyronine type	yes|down	no|up	1

G00000113581		III, opposite strand [Source:MGI Symbol;Acc:MGI:2664395]			
ENSMUSG00000060519	Tor3a	torsin family 3, member A [Source:MGI Symbol;Acc:MGI:1353652]	yes|up	no|down	1
ENSMUSG00000038143	Stox2	storkhead box 2 [Source:MGI Symbol;Acc:MGI:1918319]	yes|up	no|down	1
ENSMUSG00000085834	Gm15622	predicted gene 15622 [Source:MGI Symbol;Acc:MGI:3783067]	yes|up	no|down	1
ENSMUSG00000026839	Upp2	uridine phosphorylase 2 [Source:MGI Symbol;Acc:MGI:1923904]	yes|down	no|up	1
ENSMUSG00000029752	Asns	asparagine synthetase [Source:MGI Symbol;Acc:MGI:1350929]	yes|up	no|up	1
ENSMUSG00000072849	Serpina1e	serine (or cysteine) peptidase inhibitor, clade A, member 1E [Source:MGI Symbol;Acc:MGI:891967]	yes|up	no|up	1
ENSMUS	Prss2	protease, serine 2 [Source:MGI	yes|up	no|down	1

G00000057163		Symbol;Acc:MGI:102759]			
ENSMUSG00000093954	Gm16867	predicted gene, 16867 [Source:MGI Symbol;Acc:MGI:4439791]	yes|down	no|up	1
ENSMUSG00000022474	Pmm1	phosphomannomutase 1 [Source:MGI Symbol;Acc:MGI:1353418]	yes|up	no|down	1
ENSMUSG00000037139	Myom3	myomesin family, member 3 [Source:MGI Symbol;Acc:MGI:2685280]	yes|up	no|down	1
ENSMUSG00000055313	Pgbd1	piggyBac transposable element derived 1 [Source:MGI Symbol;Acc:MGI:2441675]	no|up	yes|down	1
ENSMUSG00000041567	Serpina12	serine (or cysteine) peptidase inhibitor, clade A (alpha-1 antiproteinase, antitrypsin), member 12 [Source:MGI Symbol;Acc:MGI:1915304]	yes|down	no|up	1
ENSMUSG00000025757	Hspa4l	heat shock protein 4 like [Source:MGI Symbol;Acc:MGI:107422]	yes|up	no|down	1

ENSMUSG00000063011	Msln	mesothelin [Source:MGI Symbol;Acc:MGI:1888992]	yes|down	no|up	1
ENSMUSG00000006777	Krt23	keratin 23 [Source:MGI Symbol;Acc:MGI:2148866]	yes|up	no|down	1
ENSMUSG00000020593	Lpin1	lipin 1 [Source:MGI Symbol;Acc:MGI:1891340]	no|up	yes|down	1
ENSMUSG00000047910	Pcdhb16	protocadherin beta 16 [Source:MGI Symbol;Acc:MGI:2136752]	no|down	yes|up	1
ENSMUSG00000020623	Map2k6	mitogen-activated protein kinase kinase 6 [Source:MGI Symbol;Acc:MGI:1346870]	yes|down	no|up	1
ENSMUSG00000040093	Bmf	BCL2 modifying factor [Source:MGI Symbol;Acc:MGI:2176433]	yes|down	no|up	1
ENSMUSG00000002068	Ccne1	cyclin E1 [Source:MGI Symbol;Acc:MGI:88316]	yes|down	no|down	1
ENSMUS	Lad1	ladinin [Source:MGI	yes|up	no|down	1

G0000004 1782		Symbol;Acc:MGI:109343]			
ENSMUS G0000010 6734	Gm205 59	predicted gene, 20559 [Source:MGI Symbol;Acc:MGI:5295666]	yes|down	yes|up	2
ENSMUS G0000005 6666	Retsat	retinol saturase (all trans retinol 13,14 reductase) [Source:MGI Symbol;Acc:MGI:1914692]	yes|up	no|down	1
ENSMUS G0000002 7654	Fam83 d	family with sequence similarity 83, member D [Source:MGI Symbol;Acc:MGI:1919128]	yes|down	no|down	1
		ENSMUSG00002076697	yes|down	no|up	1
ENSM USG0 00000 37411	Serp ine1	serine (or cysteine) peptidase inhibitor, clade E, member 1 [Source:MGI Symbol;Acc:MGI:97608]	no|up	yes|down	1
ENSM USG0 00001 03897	Pcd hga 8	protocadherin gamma subfamily A, 8 [Source:MGI Symbol;Acc:MGI:1935221]	no|down	yes|up	1
ENSM USG0	Ccn f	cyclin F [Source:MGI Symbol;Acc:MGI:102551]	yes|up	no|down	1

0000072082					
ENSMUSG0000115919	Gm31583	predicted gene, 31583 [Source:MGI Symbol;Acc:MGI:5590742]	yes|up	yes|up	2
ENSMUSG0000015653	Steap2	six transmembrane epithelial antigen of prostate 2 [Source:MGI Symbol;Acc:MGI:1921301]	yes|down	no|up	1
ENSMUSG0000026259	Ngef	neuronal guanine nucleotide exchange factor [Source:MGI Symbol;Acc:MGI:1858414]	yes|down	no|up	1
ENSMUSG0000063458	Lrmda	leucine rich melanocyte differentiation associated [Source:MGI Symbol;Acc:MGI:1923883]	yes|down	no|up	1
ENSMUSG0000001467	Cyp51	cytochrome P450, family 51 [Source:MGI Symbol;Acc:MGI:106040]	yes|up	no|down	1

ENSMUSG0000113528	Gm32699	predicted gene, 32699 [Source:MGI Symbol;Acc:MGI:5591858]	yes|down	no|no change	1
ENSMUSG0000030278	Cidec	cell death-inducing DFFA-like effector c [Source:MGI Symbol;Acc:MGI:95585]	yes|up	no|down	1
ENSMUSG0000022388	Ttll8	tubulin tyrosine ligase-like family, member 8 [Source:MGI Symbol;Acc:MGI:1922902]	no|up	yes|down	1
ENSMUSG0000083500	Gm15470	predicted gene 15470 [Source:MGI Symbol;Acc:MGI:3641891]	yes|down	no|up	1
ENSMUSG0000006517	Mvd	mevalonate (diphospho) decarboxylase [Source:MGI Symbol;Acc:MGI:2179327]	yes|up	no|down	1
ENSMUSG0	Iglv1	immunoglobulin lambda variable 1 [Source:MGI Symbol;Acc:MGI:96530]	yes|down	no|up	1

0000076934					
ENSMUSG0000115289	Gm49164	predicted gene, 49164 [Source:MGI Symbol;Acc:MGI:6118590]	no|down	yes|up	1
ENSMUSG0000113831	Gm49602	predicted gene, 49602 [Source:MGI Symbol;Acc:MGI:6215013]	yes|down	no|up	1
ENSMUSG0000032080	Apoa4	apolipoprotein A-IV [Source:MGI Symbol;Acc:MGI:88051]	yes|down	no|up	1
ENSMUSG0000031712	Il15	interleukin 15 [Source:MGI Symbol;Acc:MGI:103014]	yes|down	no|up	1
ENSMUSG0000031808	Slc27a1	solute carrier family 27 (fatty acid transporter), member 1 [Source:MGI Symbol;Acc:MGI:1347098]	yes|up	no|up	1

ENSMUSG0000041945	Mfsd9	major facilitator superfamily domain containing 9 [Source:MGI Symbol;Acc:MGI:2443548]	no|up	yes|down	1
ENSMUSG0000117499	Gm50010	predicted gene, 50010 [Source:MGI Symbol;Acc:MGI:6275297]	yes|down	no|up	1
ENSMUSG00001105204	Gm43738	predicted gene 43738 [Source:MGI Symbol;Acc:MGI:5663875]	yes|up	yes|down	2
ENSMUSG0000053461	Hhipl2	hedgehog interacting protein-like 2 [Source:MGI Symbol;Acc:MGI:1926022]	yes|up	no|down	1
ENSMUSG0000038768	9130409I23Rik	RIKEN cDNA 9130409I23 gene [Source:MGI Symbol;Acc:MGI:3588271]	yes|up	no|down	1
ENSMUSG0	Cerkl	ceramide kinase-like [Source:MGI Symbol;Acc:MGI:3037816]	yes|up	no|up	1

0000075256					
ENSMUSG0000046818	Ddit4l	DNA-damage-inducible transcript 4-like [Source:MGI Symbol;Acc:MGI:1920534]	no|down	yes|up	1
ENSMUSG0000087267	4933427J07Rik	RIKEN cDNA 4933427J07 gene [Source:MGI Symbol;Acc:MGI:1918470]	no|down	yes|up	1
ENSMUSG0000078817	Nlrp12	NLR family, pyrin domain containing 12 [Source:MGI Symbol;Acc:MGI:2676630]	yes|up	no|up	1
ENSMUSG0000113393	Gm48415	predicted gene, 48415 [Source:MGI Symbol;Acc:MGI:6097908]	yes|down	no|up	1
ENSMUSG0000057722	Lepr	leptin receptor [Source:MGI Symbol;Acc:MGI:104993]	yes|down	no|up	1

ENSMUSG0000023122	Sult1c2	sulfotransferase family, cytosolic, 1C, member 2 [Source:MGI Symbol;Acc:MGI:1916333]	yes|down	no|up	1
ENSMUSG0000083817	Gm14400	predicted gene 14400 [Source:MGI Symbol;Acc:MGI:3650076]	yes|down	no|up	1
ENSMUSG0000053168	9030619P08Rik	RIKEN cDNA 9030619P08 gene [Source:MGI Symbol;Acc:MGI:3612405]	yes|down	no|up	1
ENSMUSG0000107390	Gm43323	predicted gene 43323 [Source:MGI Symbol;Acc:MGI:5663460]	no|down	yes|up	1
ENSMUSG0000105446	Gm42605	predicted gene 42605 [Source:MGI Symbol;Acc:MGI:5662742]	yes|down	no|up	1
ENSM	H3c	H3 clustered histone 14 [Source:MGI	yes|up	no|up	1

USG000000093769	14	Symbol;Acc:MGI:2448355]			
ENSMUSG000000024029	Tff3	trefoil factor 3, intestinal [Source:MGI Symbol;Acc:MGI:104638]	yes|down	yes|up	2
ENSMUSG000000057836	Xlr3a	X-linked lymphocyte-regulated 3A [Source:MGI Symbol;Acc:MGI:109506]	yes|up	no|down	1
ENSMUSG000000074165	Zfp788	zinc finger protein 788 [Source:MGI Symbol;Acc:MGI:1914857]	yes|down	no|up	1
ENSMUSG000000093507	Gm20627	predicted gene 20627 [Source:MGI Symbol;Acc:MGI:5313074]	no|down	yes|up	1
ENSMUSG00000	Tgtp1	T cell specific GTPase 1 [Source:MGI Symbol;Acc:MGI:98734]	yes|down	no|up	1

78922					
ENSMUSG0000029245	Epha5	Eph receptor A5 [Source:MGI Symbol;Acc:MGI:99654]	yes|down	yes|up	2
ENSMUSG0000058579	Cela2a	chymotrypsin-like elastase family, member 2A [Source:MGI Symbol;Acc:MGI:95316]	yes|up	yes|down	2
ENSMUSG0000043639	Rbm20	RNA binding motif protein 20 [Source:MGI Symbol;Acc:MGI:1920963]	yes|down	no|up	1
ENSMUSG0000074398	Gm15441	predicted gene 15441 [Source:MGI Symbol;Acc:MGI:3641753]	yes|up	no|down	1
ENSMUSG0000028712	Cyp4a31	cytochrome P450, family 4, subfamily a, polypeptide 31 [Source:MGI Symbol;Acc:MGI:3028580]	yes|up	no|up	1
ENSM	Cyp	cytochrome P450, family 4, subfamily	yes|up	no|down	1

USG000000028715	4a14	a, polypeptide 14 [Source:MGI Symbol;Acc:MGI:1096550]			
ENSMUSG000000005267	Zfp287	zinc finger protein 287 [Source:MGI Symbol;Acc:MGI:2176561]	yes|down	no|up	1
ENSMUSG000000043633	Fam221b	family with sequence similarity 221, member B [Source:MGI Symbol;Acc:MGI:2441678]	yes|down	no|up	1
ENSMUSG000000024987	Cyp26a1	cytochrome P450, family 26, subfamily a, polypeptide 1 [Source:MGI Symbol;Acc:MGI:1096359]	yes|down	no|down	1
ENSMUSG000000024986	Hhex	hematopoietically expressed homeobox [Source:MGI Symbol;Acc:MGI:96086]	no|down	yes|up	1
ENSMUSG00000	Slc16a1	solute carrier family 16 (monocarboxylic acid transporters), member 1 [Source:MGI	yes|up	no|down	1

32902		Symbol;Acc:MGI:106013]			
ENSMUSG000000057110	Cntr1	centriolin [Source:MGI Symbol;Acc:MGI:1889576]	yes|up	no|down	1
ENSMUSG000000033597	Caskin1	CASK interacting protein 1 [Source:MGI Symbol;Acc:MGI:2442952]	no|down	yes|up	1
ENSMUSG000000047953	Gp5	glycoprotein 5 (platelet) [Source:MGI Symbol;Acc:MGI:1096363]	no|down	yes|up	1
ENSMUSG000000025003	Cyp2c39	cytochrome P450, family 2, subfamily c, polypeptide 39 [Source:MGI Symbol;Acc:MGI:1306818]	yes|up	no|down	1
ENSMUSG000000025002	Cyp2c55	cytochrome P450, family 2, subfamily c, polypeptide 55 [Source:MGI Symbol;Acc:MGI:1919332]	yes|down	no|up	1
ENSM	Zfp	zinc finger protein 980 [Source:MGI	yes|down	yes|up	2

USG000000058186	980	Symbol;Acc:MGI:3712454]			
ENSMUSG000000070490	Gm10293	predicted pseudogene 10293 [Source:MGI Symbol;Acc:MGI:3704216]	no|up	yes|down	1
ENSMUSG000000005413	Hmox1	heme oxygenase 1 [Source:MGI Symbol;Acc:MGI:96163]	yes|up	no|down	1
ENSMUSG000000030483	Cyp2b10	cytochrome P450, family 2, subfamily b, polypeptide 10 [Source:MGI Symbol;Acc:MGI:88598]	yes|up	no|down	1
ENSMUSG000000093577	Gm20632	predicted gene 20632 [Source:MGI Symbol;Acc:MGI:5313079]	yes|up	no|down	1
ENSMUSG00000	Gm20671	predicted gene 20671 [Source:MGI Symbol;Acc:MGI:5313118]	yes|down	no|no change	1

93574					
ENSMUSG0000051627	H1f4	H1.4 linker histone, cluster member [Source:MGI Symbol;Acc:MGI:1931527]	yes|up	no|down	1
ENSMUSG0000108207	1810059H22Rik	RIKEN cDNA 1810059H22 gene [Source:MGI Symbol;Acc:MGI:1917070]	yes|down	no|up	1
ENSMUSG0000020175	Rab36	RAB36, member RAS oncogene family [Source:MGI Symbol;Acc:MGI:1924127]	yes|up	no|down	1
ENSMUSG0000042985	Upk3b	uroplakin 3B [Source:MGI Symbol;Acc:MGI:2140882]	yes|down	yes|up	2
ENSMUSG0000020037	Rfx4	regulatory factor X, 4 (influences HLA class II expression) [Source:MGI Symbol;Acc:MGI:1918387]	yes|up	no|down	1

ENSMUSG00000018395	Kif3a	kinesin family member 3A [Source:MGI Symbol;Acc:MGI:107689]	yes|up	no|down	1
ENSMUSG00000050445	Cyp8b1	cytochrome P450, family 8, subfamily b, polypeptide 1 [Source:MGI Symbol;Acc:MGI:1338044]	yes|up	no|down	1
ENSMUSG00000045775	Slc16a5	solute carrier family 16 (monocarboxylic acid transporters), member 5 [Source:MGI Symbol;Acc:MGI:2443515]	yes|up	yes|down	2
ENSMUSG00001015497	Gm49207	predicted gene, 49207 [Source:MGI Symbol;Acc:MGI:6118657]	yes|down	yes|up	2
ENSMUSG00000004730	Adgre1	adhesion G protein-coupled receptor E1 [Source:MGI Symbol;Acc:MGI:106912]	no|up	yes|down	1
ENSMUSG0	Gm979	predicted gene 9796 [Source:MGI Symbol;Acc:MGI:3648412]	no|up	yes|down	1

0000045075	6				
ENSMUSG0000020778	Ten1	TEN1 telomerase capping complex subunit [Source:MGI Symbol;Acc:MGI:1916785]	yes|up	no|up	1
ENSMUSG0000032053	Pou2af1	POU domain, class 2, associating factor 1 [Source:MGI Symbol;Acc:MGI:105086]	no|up	yes|down	1
ENSMUSG0000026064	Ptp4a1	protein tyrosine phosphatase 4a1 [Source:MGI Symbol;Acc:MGI:1277096]	yes|down	no|down	1
ENSMUSG0000053541	Gvin-ps6	GTPase, very large interferon inducible, pseudogene 6 [Source:MGI Symbol;Acc:MGI:3647753]	yes|down	no|up	1
ENSMUSG0000074345	Tnfaip8l3	tumor necrosis factor, alpha-induced protein 8-like 3 [Source:MGI Symbol;Acc:MGI:2685363]	yes|down	no|up	1

ENSMUSG00001103585	Pcdhgb4	protocadherin gamma subfamily B, 4 [Source:MGI Symbol;Acc:MGI:1935173]	no|down	yes|up	1
ENSMUSG00000021270	Hsp90aa1	heat shock protein 90, alpha (cytosolic), class A member 1 [Source:MGI Symbol;Acc:MGI:96250]	yes|up	no|down	1
ENSMUSG00001109168	Gm44709	predicted gene 44709 [Source:MGI Symbol;Acc:MGI:5753285]	no|down	yes|up	1
ENSMUSG00000055653	Gpc3	glypican 3 [Source:MGI Symbol;Acc:MGI:104903]	yes|down	no|up	1
ENSMUSG00000067928	Zfp760	zinc finger protein 760 [Source:MGI Symbol;Acc:MGI:2679257]	no|down	yes|up	1
ENSMUSG0	Gm289	predicted gene 28959 [Source:MGI Symbol;Acc:MGI:5579665]	yes|down	no|up	1

00001 00039	59				
ENSMUSG0000100106517	Gm42634	predicted gene 42634 [Source:MGI Symbol;Acc:MGI:5662771]	yes|down	no|up	1
ENSMUSG0000000085642	3110053B16Rik	RIKEN cDNA 3110053B16 gene [Source:MGI Symbol;Acc:MGI:1920435]	no|up	yes|down	1
ENSMUSG0000000087382	Ctcflos	CCCTC-binding factor (zinc finger protein)-like, opposite strand [Source:MGI Symbol;Acc:MGI:1921411]	yes|up	no|up	1
ENSMUSG0000000031604	Msmo1	methylsterol monoxygenase 1 [Source:MGI Symbol;Acc:MGI:1913484]	yes|up	no|down	1
ENSMUSG000000	Cdk3	cyclin-dependent kinase 3 [Source:MGI Symbol;Acc:MGI:1916931]	no|down	yes|up	1

92300					
ENSMUSG00000023044	Csad	cysteine sulfinic acid decarboxylase [Source:MGI Symbol;Acc:MGI:2180098]	yes|up	no|down	1
ENSMUSG00000045551	Fpr1	formyl peptide receptor 1 [Source:MGI Symbol;Acc:MGI:107443]	no|up	yes|down	1
ENSMUSG00000023046	Igfbp6	insulin-like growth factor binding protein 6 [Source:MGI Symbol;Acc:MGI:96441]	yes|down	yes|up	2
ENSMUSG00000002732	Fkbp7	FK506 binding protein 7 [Source:MGI Symbol;Acc:MGI:1336879]	yes|down	no|up	1
ENSMUSG00000003555	Cyp17a1	cytochrome P450, family 17, subfamily a, polypeptide 1 [Source:MGI Symbol;Acc:MGI:88586]	yes|down	no|up	1
ENSM	Bm	bone morphogenetic protein 4	yes|down	no|up	1

USG00000021835	p4	[Source:MGI Symbol;Acc:MGI:88180]			
ENSMUSG00000111709	Gm3776	predicted gene 3776 [Source:MGI Symbol;Acc:MGI:3826440]	no|up	yes|down	1
ENSMUSG00000073771	Btbd19	BTB (POZ) domain containing 19 [Source:MGI Symbol;Acc:MGI:1925861]	yes|down	no|up	1
ENSMUSG00000070425	Xntrpc	Xndc1-transient receptor potential cation channel, subfamily C, member 2 readthrough [Source:MGI Symbol;Acc:MGI:5546370]	yes|up	no|down	1
ENSMUSG00000063730	Hsd3b2	hydroxy-delta-5-steroid dehydrogenase, 3 beta- and steroid delta-isomerase 2 [Source:MGI Symbol;Acc:MGI:96234]	yes|down	no|up	1
ENSMUSG000000	Zfp945	zinc finger protein 945 [Source:MGI Symbol;Acc:MGI:2445132]	yes|down	no|up	1

59142					
ENSMUSG0000021187	Tc2n	tandem C2 domains, nuclear [Source:MGI Symbol;Acc:MGI:1921663]	yes|down	no|up	1
ENSMUSG0000081205	Gm5940	predicted gene 5940 [Source:MGI Symbol;Acc:MGI:3648847]	no|up	yes|down	1
ENSMUSG0000057897	Camk2b	calcium/calmodulin-dependent protein kinase II, beta [Source:MGI Symbol;Acc:MGI:88257]	yes|up	yes|down	2
ENSMUSG0000019577	Pdk4	pyruvate dehydrogenase kinase, isoenzyme 4 [Source:MGI Symbol;Acc:MGI:1351481]	yes|up	no|down	1
ENSMUSG0000094921	Vmn2r112	vomeronasal 2, receptor 112 [Source:MGI Symbol;Acc:MGI:3644292]	no|up	yes|up	1
ENSM	Syn	synaptopodin [Source:MGI	yes|up	no|down	1

USG000000043079	po	Symbol;Acc:MGI:1099446]			
ENSMUSG000000113294	Gm49326	predicted gene, 49326 [Source:MGI Symbol;Acc:MGI:6121511]	no|up	yes|down	1
ENSMUSG00000026315	Serpinb8	serine (or cysteine) peptidase inhibitor, clade B, member 8 [Source:MGI Symbol;Acc:MGI:894657]	yes|down	no|up	1
ENSMUSG00000073835	Mup-ps12	major urinary protein, pseudogene 12 [Source:MGI Symbol;Acc:MGI:3783148]	no|up	yes|up	1
ENSMUSG00000032221	Mns1	meiosis-specific nuclear structural protein 1 [Source:MGI Symbol;Acc:MGI:107933]	no|up	yes|down	1
ENSMUSG00001	Gm10599	predicted pseudogene 10599 [Source:MGI Symbol;Acc:MGI:3710582]	yes|down	no|up	1

18458					
ENSMUSG0000079501	Gm5138	predicted gene 5138 [Source:MGI Symbol;Acc:MGI:3779464]	yes|up	no|down	1
ENSMUSG0000040229	Gpr34	G protein-coupled receptor 34 [Source:MGI Symbol;Acc:MGI:1346334]	yes|down	no|up	1
ENSMUSG0000079507	H2-Q1	histocompatibility 2, Q region locus 1 [Source:MGI Symbol;Acc:MGI:95928]	yes|down	no|down	1
ENSMUSG0000037887	Dusp8	dual specificity phosphatase 8 [Source:MGI Symbol;Acc:MGI:106626]	no|up	yes|down	1
ENSMUSG0000076617	Ighm	immunoglobulin heavy constant mu [Source:MGI Symbol;Acc:MGI:96448]	no|up	yes|down	1
ENSM	Ighg	immunoglobulin heavy constant gamma	yes|down	no|up	1

USG000000076612	2c	2C [Source:MGI Symbol;Acc:MGI:2686979]			
ENSMUSG000000076613	Ighg2b	immunoglobulin heavy constant gamma 2B [Source:MGI Symbol;Acc:MGI:96445]	yes|down	no|up	1
ENSMUSG000000056300	Zfp981	zinc finger protein 981 [Source:MGI Symbol;Acc:MGI:3700965]	no|up	yes|down	1
ENSMUSG000000067038	Rps12-ps3	ribosomal protein S12, pseudogene 3 [Source:MGI Symbol;Acc:MGI:3704503]	no|down	yes|up	1
ENSMUSG000000026818	Cel	carboxyl ester lipase [Source:MGI Symbol;Acc:MGI:88374]	yes|up	yes|down	2
ENSMUSG000000	Igkv3-4	immunoglobulin kappa variable 3-4 [Source:MGI Symbol;Acc:MGI:1330855]	yes|down	no|no change	1

96715					
ENSMUSG0000115856	Gm18095	predicted gene, 18095 [Source:MGI Symbol;Acc:MGI:5010280]	no|down	yes|up	1
ENSMUSG0000058542	Gm15590	predicted gene 15590 [Source:MGI Symbol;Acc:MGI:3831433]	no|down	yes|up	1
ENSMUSG0000057093	Zfp607b	zinc finger protein 607B [Source:MGI Symbol;Acc:MGI:2148237]	no|down	yes|up	1
ENSMUSG0000083672	Kpna2-ps	Kpna2 retrotransposed pseudogene [Source:MGI Symbol;Acc:MGI:3647335]	no|up	yes|down	1
ENSMUSG0000079165	Sap25	sin3 associated polypeptide [Source:MGI Symbol;Acc:MGI:3802945]	no|down	yes|up	1
ENSM	Pcd	protocadherin gamma subfamily A, 12	yes|down	no|up	1

USG000000102428	hga12	[Source:MGI Symbol;Acc:MGI:1935229]			
ENSMUSG0000089694	Nat8f7	N-acetyltransferase 8 (GCN5-related) family member 7 [Source:MGI Symbol;Acc:MGI:3782661]	yes|down	no|down	1
ENSMUSG0000092008	Cyp2c69	cytochrome P450, family 2, subfamily c, polypeptide 69 [Source:MGI Symbol;Acc:MGI:3721049]	yes|down	yes|up	2
ENSMUSG000000106229	Gm19409	predicted gene, 19409 [Source:MGI Symbol;Acc:MGI:5011594]	yes|up	no|down	1
ENSMUSG0000041773	Enc1	ectodermal-neural cortex 1 [Source:MGI Symbol;Acc:MGI:109610]	yes|up	no|down	1
ENSMUSG000000	Nr2c2	nuclear receptor subfamily 2, group C, member 2 [Source:MGI Symbol;Acc:MGI:1352466]	no|up	yes|down	1

05893					
ENSMUSG0000038602	Slc35f1	solute carrier family 35, member F1 [Source:MGI Symbol;Acc:MGI:2139810]	yes|up	no|up	1
ENSMUSG0000068877	Selenbp2	selenium binding protein 2 [Source:MGI Symbol;Acc:MGI:104859]	yes|up	no|up	1
ENSMUSG0000084771	A230072E10Rik	RIKEN cDNA A230072E10 gene [Source:MGI Symbol;Acc:MGI:3603753]	yes|up	no|down	1
ENSMUSG0000097431	Gm26782	predicted gene, 26782 [Source:MGI Symbol;Acc:MGI:5477276]	yes|up	no|down	1
ENSMUSG0000112796	Gm40770	predicted gene, 40770 [Source:MGI Symbol;Acc:MGI:5623655]	yes|down	yes|up	2

ENSMUSG000000064032	Gm10143	predicted gene 10143 [Source:MGI Symbol;Acc:MGI:3704492]	yes|down	no|up	1
ENSMUSG000000028008	Asic5	acid-sensing (proton-gated) ion channel family member 5 [Source:MGI Symbol;Acc:MGI:1929259]	yes|up	no|down	1
ENSMUSG000001111890	Gm48836	predicted gene, 48836 [Source:MGI Symbol;Acc:MGI:6098565]	yes|down	no|up	1
ENSMUSG000000035934	Pknox2	Pbx/knotted 1 homeobox 2 [Source:MGI Symbol;Acc:MGI:2445415]	yes|up	no|down	1
ENSMUSG000000035686	Thrsp	thyroid hormone responsive [Source:MGI Symbol;Acc:MGI:109126]	yes|down	no|down	1
ENSMUSG0	Per3	period circadian clock 3 [Source:MGI Symbol;Acc:MGI:1277134]	yes|down	no|up	1

0000028957					
ENSMUSG0000070568	Slc6a21	solute carrier family 6 member 21 [Source:MGI Symbol;Acc:MGI:1923963]	yes|up	no|up	1
ENSMUSG0000070605	Zfp992	zinc finger protein 992 [Source:MGI Symbol;Acc:MGI:3700963]	no|down	yes|up	1
ENSMUSG0000026639	Lamb3	laminin, beta 3 [Source:MGI Symbol;Acc:MGI:99915]	yes|up	no|down	1
ENSMUSG0000094433	Igkv5-43	immunoglobulin kappa chain variable 5-43 [Source:MGI Symbol;Acc:MGI:4943320]	yes|up	no|down	1
ENSMUSG0000018900	Slc22a5	solute carrier family 22 (organic cation transporter), member 5 [Source:MGI Symbol;Acc:MGI:1329012]	yes|up	no|down	1

ENSMUSG0000072949	Acot1	acyl-CoA thioesterase 1 [Source:MGI Symbol;Acc:MGI:1349396]	yes|up	no|down	1
ENSMUSG0000019817	Plagl1	pleiomorphic adenoma gene-like 1 [Source:MGI Symbol;Acc:MGI:1100874]	yes|down	no|up	1
ENSMUSG0000086370	Ftx	Ftx transcript, Xist regulator (non-protein coding) [Source:MGI Symbol;Acc:MGI:1926128]	yes|down	no|up	1
ENSMUSG0000024430	Cabyr	calcium-binding tyrosine-(Y)-phosphorylation regulated (fibrousheathin 2) [Source:MGI Symbol;Acc:MGI:1918382]	yes|down	no|up	1
ENSMUSG0000105192	Gm42796	predicted gene 42796 [Source:MGI Symbol;Acc:MGI:5662933]	yes|down	yes|up	2
ENSMUSG0	Syn3	synapsin III [Source:MGI Symbol;Acc:MGI:1351334]	no|down	yes|up	1

0000059602					
ENSMUSG00000002289	Angptl4	angiopoietin-like 4 [Source:MGI Symbol;Acc:MGI:1888999]	yes|up	no|down	1
ENSMUSG00000004651	Tyr	tyrosinase [Source:MGI Symbol;Acc:MGI:98880]	no|down	yes|up	1
ENSMUSG00000027035	Cers6	ceramide synthase 6 [Source:MGI Symbol;Acc:MGI:2442564]	yes|down	no|up	1
ENSMUSG00001106702	Gm42846	predicted gene 42846 [Source:MGI Symbol;Acc:MGI:5662983]	no|down	yes|down	1
ENSMUSG00000030545	Pex11a	peroxisomal biogenesis factor 11 alpha [Source:MGI Symbol;Acc:MGI:1338788]	yes|up	no|down	1

ENSMUSG0000032363	Adamts7	a disintegrin-like and metallopeptidase (reprolysin type) with thrombospondin type 1 motif, 7 [Source:MGI Symbol;Acc:MGI:1347346]	yes|down	no|up	1
ENSMUSG0000017057	Il13ra1	interleukin 13 receptor, alpha 1 [Source:MGI Symbol;Acc:MGI:105052]	yes|down	no|up	1
ENSMUSG0000020642	Rnf144a	ring finger protein 144A [Source:MGI Symbol;Acc:MGI:1344401]	yes|up	no|down	1
ENSMUSG0000106863	Gm42109	predicted gene, 42109 [Source:MGI Symbol;Acc:MGI:5624994]	yes|down	no|up	1
ENSMUSG0000082381	Gm12928	predicted gene 12928 [Source:MGI Symbol;Acc:MGI:3652022]	no|up	yes|down	1
ENSMUSG0	Cnmd	chondromodulin [Source:MGI Symbol;Acc:MGI:1341171]	yes|down	no|up	1

0000022025					
ENSMUSG00000094152	Slc6a16	solute carrier family 6, member 16 [Source:MGI Symbol;Acc:MGI:2685930]	yes|up	no|down	1
ENSMUSG00000051225	Fam83a	family with sequence similarity 83, member A [Source:MGI Symbol;Acc:MGI:2447773]	no|up	yes|down	1
ENSMUSG00000048905	Bnip5	BCL2 interacting protein 5 [Source:MGI Symbol;Acc:MGI:1925441]	yes|up	no|down	1
ENSMUSG00001115022	Gm49387	predicted gene, 49387 [Source:MGI Symbol;Acc:MGI:6121612]	yes|down	yes|up	2
ENSMUSG00001118012	Gm46620	predicted gene, 46620 [Source:MGI Symbol;Acc:MGI:5826257]	yes|up	yes|down	2

ENSMUSG0000031637	Lrp2bp	Lrp2 binding protein [Source:MGI Symbol;Acc:MGI:1914870]	yes|down	no|up	1
ENSMUSG0000096056	Gm21986	predicted gene 21986 [Source:MGI Symbol;Acc:MGI:5439455]	no|up	yes|down	1
ENSMUSG00000104986	1600023N17Rik	RIKEN cDNA 1600023N17 gene [Source:MGI Symbol;Acc:MGI:1917038]	no|down	yes|up	1
ENSMUSG0000099032	Tcf24	transcription factor 24 [Source:MGI Symbol;Acc:MGI:3780500]	yes|down	no|up	1
ENSMUSG0000044349	Snhg11	small nucleolar RNA host gene 11 [Source:MGI Symbol;Acc:MGI:2441845]	yes|down	no|up	1
ENSM	170	RIKEN cDNA 1700018L02 gene	no|down	yes|up	1

USG0 00001 00075	001 8L0 2Ri k	[Source:MGI Symbol;Acc:MGI:1914579]			
ENSM USG0 00000 32500	Dcl k3	doublecortin-like kinase 3 [Source:MGI Symbol;Acc:MGI:3039580]	yes|down	no|up	1
ENSM USG0 00000 11305	Plin 5	perilipin 5 [Source:MGI Symbol;Acc:MGI:1914218]	yes|up	no|down	1
ENSM USG0 00000 60548	Tnfr sf19	tumor necrosis factor receptor superfamily, member 19 [Source:MGI Symbol;Acc:MGI:1352474]	yes|down	no|up	1
ENSM USG0 00001 10488	Gm 457 24	predicted gene 45724 [Source:MGI Symbol;Acc:MGI:5804839]	no|down	yes|up	1
ENSM USG0	BC0 259	cDNA sequence BC025920 [Source:MGI	no|up	yes|up	1

0000074862	20	Symbol;Acc:MGI:2670983]			
ENSMUSG0000021908	Ncoa4-ps	nuclear receptor coactivator 4, pseudogene [Source:MGI Symbol;Acc:MGI:3648259]	no|down	yes|up	1
ENSMUSG0000024036	Slc37a1	solute carrier family 37 (glycerol-3-phosphate transporter), member 1 [Source:MGI Symbol;Acc:MGI:2446181]	yes|down	no|down	1
ENSMUSG0000027698	Nceh1	neutral cholesterol ester hydrolase 1 [Source:MGI Symbol;Acc:MGI:2443191]	yes|up	no|down	1
ENSMUSG0000021453	Gadd45g	growth arrest and DNA-damage-inducible 45 gamma [Source:MGI Symbol;Acc:MGI:1346325]	yes|down	no|up	1
ENSMUSG0000097836	Gm26903	predicted gene, 26903 [Source:MGI Symbol;Acc:MGI:5477397]	no|down	yes|down	1

ENSMUSG000000086103	Gm11832	predicted gene 11832 [Source:MGI Symbol;Acc:MGI:3650310]	yes|up	no|down	1
ENSMUSG000000114133	Gm20075	predicted gene, 20075 [Source:MGI Symbol;Acc:MGI:5012260]	yes|down	no|up	1
ENSMUSG000000026853	Crat	carnitine acetyltransferase [Source:MGI Symbol;Acc:MGI:109501]	yes|up	no|down	1
ENSMUSG000000036938	Try5	trypsin 5 [Source:MGI Symbol;Acc:MGI:102756]	no|up	yes|down	1
ENSMUSG000000038415	Foxq1	forkhead box Q1 [Source:MGI Symbol;Acc:MGI:1298228]	yes|down	yes|up	2
ENSMUSG0	Trpc2	transient receptor potential cation channel, subfamily C, member 2	yes|down	no|up	1

0000100254		[Source:MGI Symbol;Acc:MGI:109527]			
ENSMUSG0000078937	Cpt1b	carnitine palmitoyltransferase 1b, muscle [Source:MGI Symbol;Acc:MGI:1098297]	yes|up	no|up	1
ENSMUSG0000038418	Egr1	early growth response 1 [Source:MGI Symbol;Acc:MGI:95295]	yes|up	no|down	1
ENSMUSG0000106705	Gm2602	predicted gene 2602 [Source:MGI Symbol;Acc:MGI:3780770]	yes|down	no|up	1
ENSMUSG0000022351	Sqle	squalene epoxidase [Source:MGI Symbol;Acc:MGI:109296]	yes|up	yes|down	2
ENSMUSG0000044645	Gm7334	predicted gene 7334 [Source:MGI Symbol;Acc:MGI:3647393]	yes|up	no|down	1

ENSMUSG00000073842	Mup7	major urinary protein 7 [Source:MGI Symbol;Acc:MGI:3709615]	yes|up	no|up	1
ENSMUSG00000053706	B430305J03Rik	RIKEN cDNA B430305J03 gene [Source:MGI Symbol;Acc:MGI:3697707]	no|up	yes|down	1
ENSMUSG00000028871	Rspo1	R-spondin 1 [Source:MGI Symbol;Acc:MGI:2183426]	yes|down	no|up	1
ENSMUSG00000090942	F830016B08Rik	RIKEN cDNA F830016B08 gene [Source:MGI Symbol;Acc:MGI:3588218]	yes|down	no|no change	1
ENSMUSG00000061451	Tmem151a	transmembrane protein 151A [Source:MGI Symbol;Acc:MGI:2147713]	yes|down	no|up	1
ENSM	Bcl6	B cell leukemia/lymphoma 6	yes|up	no|down	1

USG0000022508		[Source:MGI Symbol;Acc:MGI:107187]			
ENSMUSG0000039304	Tnfsf10	tumor necrosis factor (ligand) superfamily, member 10 [Source:MGI Symbol;Acc:MGI:107414]	yes|down	no|up	1
ENSMUSG0000025396	Hsd17b6	hydroxysteroid (17-beta) dehydrogenase 6 [Source:MGI Symbol;Acc:MGI:1351670]	yes|down	no|up	1
ENSMUSG0000081010	Gm13880	predicted gene 13880 [Source:MGI Symbol;Acc:MGI:3649539]	yes|up	no|down	1
ENSMUSG0000026675	Hsd17b7	hydroxysteroid (17-beta) dehydrogenase 7 [Source:MGI Symbol;Acc:MGI:1330808]	yes|up	no|down	1
ENSMUSG00000	Gm26608	predicted gene, 26608 [Source:MGI Symbol;Acc:MGI:5477102]	yes|up	no|up	1

97673					
ENSMUSG00000096472	Cdkn2d	cyclin dependent kinase inhibitor 2D [Source:MGI Symbol;Acc:MGI:105387]	yes|down	no|up	1
ENSMUSG00000025375	Aatk	apoptosis-associated tyrosine kinase [Source:MGI Symbol;Acc:MGI:1197518]	yes|up	no|down	1
ENSMUSG00000118631	Gm53019	predicted gene, 53019 [Source:MGI Symbol;Acc:MGI:6388910]	yes|down	no|up	1
ENSMUSG00000083332	Gm7599	predicted gene 7599 [Source:MGI Symbol;Acc:MGI:3644309]	yes|down	no|up	1
ENSMUSG00000049872	Calhm5	calcium homeostasis modulator family member 5 [Source:MGI Symbol;Acc:MGI:2143897]	yes|down	no|up	1
ENSM	Rab	RAB30, member RAS oncogene family	yes|up	no|down	1

USG000000030643	30	[Source:MGI Symbol;Acc:MGI:1923235]			
ENSMUSG0000039809	Gabbr2	gamma-aminobutyric acid (GABA) B receptor, 2 [Source:MGI Symbol;Acc:MGI:2386030]	yes|down	no|up	1
ENSMUSG0000000120	Ngfr	nerve growth factor receptor (TNFR superfamily, member 16) [Source:MGI Symbol;Acc:MGI:97323]	no|up	yes|down	1
ENSMUSG00001116347	Gm49416	predicted gene, 49416 [Source:MGI Symbol;Acc:MGI:6155046]	yes|up	no|down	1
ENSMUSG0000027210	Meis2	Meis homeobox 2 [Source:MGI Symbol;Acc:MGI:108564]	yes|down	no|up	1
ENSMUSG00000	Tuba1a	tubulin, alpha 1A [Source:MGI Symbol;Acc:MGI:98869]	yes|down	no|up	1

72235					
ENSMUSG0000001025	S100a6	S100 calcium binding protein A6 (calcyclin) [Source:MGI Symbol;Acc:MGI:1339467]	yes|down	no|up	1
ENSMUSG0000015312	Gadd45b	growth arrest and DNA-damage-inducible 45 beta [Source:MGI Symbol;Acc:MGI:107776]	yes|up	no|down	1
ENSMUSG0000004038	Gstm3	glutathione S-transferase, mu 3 [Source:MGI Symbol;Acc:MGI:106026]	yes|down	no|down	1
ENSMUSG0000040616	Tmem51	transmembrane protein 51 [Source:MGI Symbol;Acc:MGI:2384874]	yes|down	no|up	1
ENSMUSG0000074768	Bhmt	betaine-homocysteine methyltransferase [Source:MGI Symbol;Acc:MGI:1339972]	yes|up	no|up	1
ENSM	Gm	predicted gene 43798 [Source:MGI	yes|up	yes|down	2

USG000000106675	43798	Symbol;Acc:MGI:5663935]			
ENSMUSG0000062545	Tlr12	toll-like receptor 12 [Source:MGI Symbol;Acc:MGI:3045221]	yes|up	yes|down	2
ENSMUSG0000026773	Pfkfb3	6-phosphofructo-2-kinase/fructose-2,6-biphosphatase 3 [Source:MGI Symbol;Acc:MGI:2181202]	no|down	yes|down	1
ENSMUSG0000097150	Gm26513	predicted gene, 26513 [Source:MGI Symbol;Acc:MGI:5477007]	no|down	yes|up	1
ENSMUSG0000049580	Tsku	tsukushi, small leucine rich proteoglycan [Source:MGI Symbol;Acc:MGI:2443855]	yes|up	yes|down	2
ENSMUSG00000	Gucy2c	guanylate cyclase 2c [Source:MGI Symbol;Acc:MGI:106903]	yes|down	no|up	1

42638					
ENSMUSG0000046688	Tifa	TRAF-interacting protein with forkhead-associated domain [Source:MGI Symbol;Acc:MGI:2182965]	yes|down	no|up	1
ENSMUSG0000109807	Gm45244	predicted gene 45244 [Source:MGI Symbol;Acc:MGI:5791080]	no|down	yes|up	1
ENSMUSG0000108402	9430064I24Rik	RIKEN cDNA 9430064I24 gene [Source:MGI Symbol;Acc:MGI:3704302]	yes|up	no|up	1
ENSMUSG0000045404	Kcnk13	potassium channel, subfamily K, member 13 [Source:MGI Symbol;Acc:MGI:2384976]	yes|up	no|down	1
ENSMUSG0000045010	Gm4779	predicted gene 4779 [Source:MGI Symbol;Acc:MGI:3646776]	yes|down	yes|up	2
ENSM	Nbl	NBL1, DAN family BMP antagonist	yes|down	no|up	1

USG000000041120	1	[Source:MGI Symbol;Acc:MGI:104591]			
ENSMUSG000000034413	Neurl1b	neuralized E3 ubiquitin protein ligase 1B [Source:MGI Symbol;Acc:MGI:3643092]	yes|down	no|up	1
ENSMUSG000000072999	Gm15401	predicted gene 15401 [Source:MGI Symbol;Acc:MGI:3642201]	yes|up	no|up	1
ENSMUSG000000040660	Cyp2b9	cytochrome P450, family 2, subfamily b, polypeptide 9 [Source:MGI Symbol;Acc:MGI:88600]	yes|down	no|up	1
ENSMUSG000000027824	Vmn2r1	vomeronasal 2, receptor 1 [Source:MGI Symbol;Acc:MGI:3645892]	no|down	yes|up	1
ENSMUSG000000	Paqr7	progestin and adipoQ receptor family member VII [Source:MGI Symbol;Acc:MGI:1919154]	yes|up	no|down	1

37348					
ENSMUSG00000091021	Gm17300	predicted gene, 17300 [Source:MGI Symbol;Acc:MGI:4936934]	yes|down	no|up	1
ENSMUSG00000028525	Pde4b	phosphodiesterase 4B, cAMP specific [Source:MGI Symbol;Acc:MGI:99557]	yes|down	no|up	1
ENSMUSG00000040505	Abcg5	ATP binding cassette subfamily G member 5 [Source:MGI Symbol;Acc:MGI:1351659]	yes|down	no|up	1
ENSMUSG00000076498	Trbc2	T cell receptor beta, constant 2 [Source:MGI Symbol;Acc:MGI:4835227]	yes|down	yes|up	2
ENSMUSG00000032735	Ablim3	actin binding LIM protein family, member 3 [Source:MGI Symbol;Acc:MGI:2442582]	yes|up	no|down	1
ENSM	Nol	nucleolar protein 3 (apoptosis repressor	yes|down	no|up	1

USG0 00000 14776	3	with CARD domain) [Source:MGI Symbol;Acc:MGI:1925938]			
ENSMUSG0 00000 30827	Fgf21	fibroblast growth factor 21 [Source:MGI Symbol;Acc:MGI:1861377]	yes|up	no|up	1
ENSMUSG0 00000 61780	Cfd	complement factor D (adipsin) [Source:MGI Symbol;Acc:MGI:87931]	yes|up	no|down	1
ENSMUSG0 00000 78683	Mup1	major urinary protein 1 [Source:MGI Symbol;Acc:MGI:97233]	yes|up	no|up	1
ENSMUSG0 00001 18506	1700094D03Rik	RIKEN cDNA 1700094D03 gene [Source:MGI Symbol;Acc:MGI:1920795]	yes|down	no|up	1
ENSMUSG0	Lipg	lipase, endothelial [Source:MGI Symbol;Acc:MGI:1341803]	yes|up	no|down	1

0000053846					
ENSMUSG0000030218	Mgp	matrix Gla protein [Source:MGI Symbol;Acc:MGI:96976]	yes|down	no|up	1
ENSMUSG0000110397	Gm45540	predicted gene 45540 [Source:MGI Symbol;Acc:MGI:5791376]	yes|down	no|up	1
ENSMUSG0000023078	Cxcl13	chemokine (C-X-C motif) ligand 13 [Source:MGI Symbol;Acc:MGI:1888499]	yes|up	no|down	1
ENSMUSG0000082868	Mup-ps5	major urinary protein, pseudogene 5 [Source:MGI Symbol;Acc:MGI:3650601]	yes|up	no|up	1
ENSMUSG0000053830	Gm9923	predicted pseudogene 9923 [Source:MGI Symbol;Acc:MGI:3704365]	no|up	yes|down	1

ENSMUSG0000004951	Hspb1	heat shock protein 1 [Source:MGI Symbol;Acc:MGI:96240]	yes|up	no|up	1
ENSMUSG0000026390	Marco	macrophage receptor with collagenous structure [Source:MGI Symbol;Acc:MGI:1309998]	yes|up	no|down	1
ENSMUSG0000031271	Serpina7	serine (or cysteine) peptidase inhibitor, clade A (alpha-1 antiproteinase, antitrypsin), member 7 [Source:MGI Symbol;Acc:MGI:3041197]	yes|down	no|up	1
ENSMUSG0000027559	Car3	carbonic anhydrase 3 [Source:MGI Symbol;Acc:MGI:88270]	yes|down	no|down	1
ENSMUSG0000085192	Gm12195	predicted gene 12195 [Source:MGI Symbol;Acc:MGI:3651100]	yes|up	no|up	1
ENSMUSG0	H3c15	H3 clustered histone 15 [Source:MGI Symbol;Acc:MGI:2448357]	yes|up	no|down	1

0000081058					
ENSMUSG0000058258	Idi1	isopentenyl-diphosphate delta isomerase [Source:MGI Symbol;Acc:MGI:2442264]	yes|up	no|down	1
ENSMUSG0000021198	Unc79	unc-79 homolog [Source:MGI Symbol;Acc:MGI:2684729]	yes|up	no|down	1
ENSMUSG0000043068	Fam89a	family with sequence similarity 89, member A [Source:MGI Symbol;Acc:MGI:1916877]	yes|up	no|down	1
ENSMUSG0000112805	Gm34574	predicted gene, 34574 [Source:MGI Symbol;Acc:MGI:5593733]	yes|down	no|up	1
ENSMUSG0000078377	Gm4294	predicted gene 4294 [Source:MGI Symbol;Acc:MGI:3782472]	yes|down	no|up	1

ENSMUSG00001 10755	BC0 499 87	cDNA sequence BC049987 [Source:MGI Symbol;Acc:MGI:3039606]	yes|up	no|up	1
ENSMUSG00000 32724	Abt b2	ankyrin repeat and BTB (POZ) domain containing 2 [Source:MGI Symbol;Acc:MGI:2139365]	yes|up	no|down	1
ENSMUSG00000 34647	Ank rd12	ankyrin repeat domain 12 [Source:MGI Symbol;Acc:MGI:1914357]	yes|down	no|up	1
ENSMUSG00001 04660	Gm 436 01	predicted gene 43601 [Source:MGI Symbol;Acc:MGI:5663738]	yes|up	no|down	1
ENSMUSG00000 95649	Gvi n-ps 3	GTPase, very large interferon inducible, pseudogene 3 [Source:MGI Symbol;Acc:MGI:3703149]	yes|down	no|up	1
ENSM USG0	Wsb 1	WD repeat and SOCS box-containing 1 [Source:MGI	yes|down	no|up	1

0000017677		Symbol;Acc:MGI:1926139]			
ENSMUSG00000026730	Pter	phosphotriesterase related [Source:MGI Symbol;Acc:MGI:107372]	yes|down	no|up	1
ENSMUSG00000042500	Ago4	argonaute RISC catalytic subunit 4 [Source:MGI Symbol;Acc:MGI:1924100]	yes|down	no|up	1
ENSMUSG00000102091	Olfr1034	olfactory receptor 1034 [Source:MGI Symbol;Acc:MGI:3030868]	yes|down	no|up	1
ENSMUSG00000099599	Gm28548	predicted gene 28548 [Source:MGI Symbol;Acc:MGI:5579254]	yes|down	no|no change	1
ENSMUSG00000051977	Prdm9	PR domain containing 9 [Source:MGI Symbol;Acc:MGI:2384854]	yes|down	no|up	1

ENSMUSG0000094793	Mup12	major urinary protein 12 [Source:MGI Symbol;Acc:MGI:3780193]	yes|up	no|up	1
ENSMUSG0000038599	Capn8	calpain 8 [Source:MGI Symbol;Acc:MGI:2181366]	yes|up	no|up	1
ENSMUSG0000005362	Crbn	cereblon [Source:MGI Symbol;Acc:MGI:1913277]	no|up	yes|down	1
ENSMUSG0000022853	Ehhadh	enoyl-Coenzyme A, hydratase/3-hydroxyacyl Coenzyme A dehydrogenase [Source:MGI Symbol;Acc:MGI:1277964]	yes|up	no|up	1
ENSMUSG0000049124	Gm8186	predicted gene 8186 [Source:MGI Symbol;Acc:MGI:3643110]	yes|down	no|up	1
ENSMUSG0	Fzd8	frizzled class receptor 8 [Source:MGI Symbol;Acc:MGI:108460]	yes|down	no|up	1

0000036904					
ENSMUSG00000048486	Fitm2	fat storage-inducing transmembrane protein 2 [Source:MGI Symbol;Acc:MGI:2444508]	yes|up	no|down	1
ENSMUSG00000020205	Phlda1	pleckstrin homology like domain, family A, member 1 [Source:MGI Symbol;Acc:MGI:1096880]	yes|down	yes|up	2
ENSMUSG00000028597	Gpx7	glutathione peroxidase 7 [Source:MGI Symbol;Acc:MGI:1914555]	yes|up	no|down	1
ENSMUSG00000095545	Zfp969	zinc finger protein 969 [Source:MGI Symbol;Acc:MGI:3782422]	yes|down	no|up	1
ENSMUSG00000086282	Sox5os4	SRY (sex determining region Y)-box 5, opposite strand 4 [Source:MGI Symbol;Acc:MGI:3783128]	no|down	yes|up	1

ENSMUSG00000037071	Scd1	stearoyl-Coenzyme A desaturase 1 [Source:MGI Symbol;Acc:MGI:98239]	yes|down	yes|down	2
ENSMUSG00000034853	Acot11	acyl-CoA thioesterase 11 [Source:MGI Symbol;Acc:MGI:1913736]	yes|down	no|up	1
ENSMUSG00000034855	Cxcl10	chemokine (C-X-C motif) ligand 10 [Source:MGI Symbol;Acc:MGI:1352450]	yes|down	no|up	1
ENSMUSG00000027322	Siglec1	sialic acid binding Ig-like lectin 1, sialoadhesin [Source:MGI Symbol;Acc:MGI:99668]	yes|up	no|down	1
ENSMUSG00000018604	Tbx3	T-box 3 [Source:MGI Symbol;Acc:MGI:98495]	yes|up	no|down	1
ENSMUSG0	Plin2	perilipin 2 [Source:MGI Symbol;Acc:MGI:87920]	yes|up	no|down	1

0000028494					
ENSMUSG0000109473	B930025P03Rik	RIKEN cDNA B930025P03 gene [Source:MGI Symbol;Acc:MGI:2443166]	yes|up	no|up	1
ENSMUSG0000103789	Gm10129	predicted gene 10129 [Source:MGI Symbol;Acc:MGI:3641890]	yes|down	no|no change	1
ENSMUSG0000002297	Dbf4	DBF4 zinc finger [Source:MGI Symbol;Acc:MGI:1351328]	yes|down	no|down	1
ENSMUSG0000083111	Gm14421	predicted gene 14421 [Source:MGI Symbol;Acc:MGI:3652254]	yes|down	no|up	1
ENSMUSG000000	Zfp976	zinc finger protein 976 [Source:MGI Symbol;Acc:MGI:3036263]	yes|down	no|up	1

74158					
ENSMUSG0000074254	Cyp2a4	cytochrome P450, family 2, subfamily a, polypeptide 4 [Source:MGI Symbol;Acc:MGI:88596]	yes|down	no|up	1
ENSMUSG0000029882	2210010C04Rik	RIKEN cDNA 2210010C04 gene [Source:MGI Symbol;Acc:MGI:1914623]	yes|up	no|down	1
ENSMUSG0000006538	Ihh	Indian hedgehog [Source:MGI Symbol;Acc:MGI:96533]	yes|down	no|up	1
ENSMUSG0000021228	Acot3	acyl-CoA thioesterase 3 [Source:MGI Symbol;Acc:MGI:2159619]	yes|up	no|down	1
ENSMUSG0000021226	Acot2	acyl-CoA thioesterase 2 [Source:MGI Symbol;Acc:MGI:2159605]	yes|up	no|down	1

ENSMUSG00000082045	Gm11585	predicted gene 11585 [Source:MGI Symbol;Acc:MGI:3651395]	yes|down	no|up	1
ENSMUSG00000040537	Adam22	a disintegrin and metallopeptidase domain 22 [Source:MGI Symbol;Acc:MGI:1340046]	yes|down	no|down	1
ENSMUSG00000006782	Cnp	2',3'-cyclic nucleotide 3' phosphodiesterase [Source:MGI Symbol;Acc:MGI:88437]	yes|down	no|up	1
ENSMUSG00000095061	E030018B13Rik	RIKEN cDNA E030018B13 gene [Source:MGI Symbol;Acc:MGI:2686543]	yes|up	no|down	1
ENSMUSG00000023034	Nr4a1	nuclear receptor subfamily 4, group A, member 1 [Source:MGI Symbol;Acc:MGI:1352454]	yes|down	no|up	1
ENSM	Rgs	regulator of G-protein signaling 3	yes|down	no|up	1

USG000000059810	3	[Source:MGI Symbol;Acc:MGI:1354734]			
ENSMUSG000000094410	Zbed6	zinc finger, BED type containing 6 [Source:NCBI gene (formerly Entrezgene);Acc:667118]	yes|up	no|down	1
ENSMUSG000000022032	Scara5	scavenger receptor class A, member 5 [Source:MGI Symbol;Acc:MGI:1918395]	no|up	yes|down	1
ENSMUSG000000062101	Zfp119b	zinc finger protein 119b [Source:MGI Symbol;Acc:MGI:2385323]	yes|down	no|up	1
ENSMUSG000000008153	Clstn3	calsyntenin 3 [Source:MGI Symbol;Acc:MGI:2178323]	yes|up	no|down	1
ENSMUSG000000	Mpc1-ps	mitochondrial pyruvate carrier 1, pseudogene [Source:MGI Symbol;Acc:MGI:3781628]	no|down	yes|up	1

91498					
ENSMUSG0000062061	Obp2a	odorant binding protein 2A [Source:MGI Symbol;Acc:MGI:2387617]	yes|up	no|down	1
ENSMUSG0000102742	Pcdhga11	protocadherin gamma subfamily A, 11 [Source:MGI Symbol;Acc:MGI:1935228]	yes|down	no|up	1
ENSMUSG0000087611	4930458D05Rik	RIKEN cDNA 4930458D05 gene [Source:MGI Symbol;Acc:MGI:1923052]	yes|up	no|up	1
ENSMUSG0000039385	Cdh6	cadherin 6 [Source:MGI Symbol;Acc:MGI:107435]	no|down	yes|up	1
ENSMUSG0000074513	Arfip1	ADP-ribosylation factor interacting protein 1 [Source:MGI Symbol;Acc:MGI:1277120]	no|up	yes|down	1

ENSMUSG0000090264	Eif4ebp3	eukaryotic translation initiation factor 4E binding protein 3 [Source:MGI Symbol;Acc:MGI:1270847]	yes|up	no|up	1
ENSMUSG0000118471	Gm50461	predicted gene, 50461 [Source:MGI Symbol;Acc:MGI:6324726]	yes|down	no|up	1
ENSMUSG0000029161	Cgref1	cell growth regulator with EF hand domain 1 [Source:MGI Symbol;Acc:MGI:1915817]	yes|up	no|down	1
ENSMUSG0000060807	Serpina6	serine (or cysteine) peptidase inhibitor, clade A, member 6 [Source:MGI Symbol;Acc:MGI:88278]	yes|down	no|up	1
ENSMUSG0000106566	Gm43836	predicted gene 43836 [Source:MGI Symbol;Acc:MGI:5663973]	yes|up	no|down	1
ENSMUSG0	Chrna2	cholinergic receptor, nicotinic, alpha polypeptide 2 (neuronal) [Source:MGI	yes|up	no|down	1

0000022041		Symbol;Acc:MGI:87886]			
ENSMUSG0000014498	Ankrd52	ankyrin repeat domain 52 [Source:MGI Symbol;Acc:MGI:2444029]	no|up	yes|down	1
ENSMUSG0000057913	Gm10032	predicted gene 10032 [Source:MGI Symbol;Acc:MGI:3642901]	yes|up	no|down	1
ENSMUSG0000006218	Fam131c	family with sequence similarity 131, member C [Source:MGI Symbol;Acc:MGI:2685539]	yes|up	no|down	1
ENSMUSG0000078886	Gm2026	predicted gene 2026 [Source:MGI Symbol;Acc:MGI:3780195]	no|up	yes|down	1
ENSMUSG0000078881	Gm14434	predicted gene 14434 [Source:MGI Symbol;Acc:MGI:3702417]	no|down	yes|up	1

ENSMUSG00000078898	Zfp968	zinc finger protein 968 [Source:MGI Symbol;Acc:MGI:3782903]	no|down	yes|up	1
ENSMUSG00001105509	C130013H08Rik	RIKEN cDNA C130013H08 gene [Source:MGI Symbol;Acc:MGI:3697343]	yes|up	no|down	1
ENSMUSG00000099413	Gm17767	predicted gene, 17767 [Source:MGI Symbol;Acc:MGI:5009931]	yes|up	no|down	1
ENSMUSG00000092072	Gm4540	predicted gene 4540 [Source:MGI Symbol;Acc:MGI:3782724]	no|down	yes|up	1
ENSMUSG00001114123	Gm48416	predicted gene, 48416 [Source:MGI Symbol;Acc:MGI:6097909]	yes|down	yes|up	2
ENSM	Pcsk	proprotein convertase subtilisin/kexin	yes|up	yes|down	2

USG000000044254	9	type 9 [Source:MGI Symbol;Acc:MGI:2140260]			
ENSMUSG000000002831	Plin4	perilipin 4 [Source:MGI Symbol;Acc:MGI:1929709]	yes|up	no|down	1
ENSMUSG000000044098	Rsbn1	rosbin, round spermatid basic protein 1 [Source:MGI Symbol;Acc:MGI:2444993]	yes|down	no|up	1
ENSMUSG000000057777	Mab21l2	mab-21-like 2 [Source:MGI Symbol;Acc:MGI:1346022]	yes|up	no|down	1
ENSMUSG000000026185	Igfbp5	insulin-like growth factor binding protein 5 [Source:MGI Symbol;Acc:MGI:96440]	yes|down	no|up	1
ENSMUSG000000	Snhg20	small nucleolar RNA host gene 20 [Source:MGI Symbol;Acc:MGI:1924222]	yes|down	no|up	1

86859					
ENSMUSG0000058454	Dhcr7	7-dehydrocholesterol reductase [Source:MGI Symbol;Acc:MGI:1298378]	yes|up	no|down	1
ENSMUSG0000058216	Gstp3	glutathione S-transferase pi 3 [Source:MGI Symbol;Acc:MGI:2385078]	yes|down	no|up	1
ENSMUSG0000022346	Myc	myelocytomatosis oncogene [Source:MGI Symbol;Acc:MGI:97250]	no|up	yes|up	1
ENSMUSG0000079523	Tmsb10	thymosin, beta 10 [Source:MGI Symbol;Acc:MGI:109146]	yes|down	no|up	1
ENSMUSG0000059743	Fdps	farnesyl diphosphate synthetase [Source:MGI Symbol;Acc:MGI:104888]	yes|up	no|down	1
ENSM	Gm	predicted gene 45140 [Source:MGI	yes|up	no|down	1

USG000001 07928	45140	Symbol;Acc:MGI:5753716]			
ENSMUSG0000058297	Spock2	sparc/osteonectin, cwcv and kazal-like domains proteoglycan 2 [Source:MGI Symbol;Acc:MGI:1891351]	yes|down	no|up	1
ENSMUSG0000034774	Dsg1c	desmoglein 1 gamma [Source:MGI Symbol;Acc:MGI:2664358]	yes|down	no|up	1
ENSMUSG0000116508	Gm49463	predicted gene, 49463 [Source:MGI Symbol;Acc:MGI:6155122]	no|up	yes|down	1
ENSMUSG0000067158	Col4a4	collagen, type IV, alpha 4 [Source:MGI Symbol;Acc:MGI:104687]	no|down	yes|up	1
ENSMUSG000000	Gm7332	predicted gene 7332 [Source:MGI Symbol;Acc:MGI:3643344]	no|down	yes|down	1

80875					
ENSMUSG0000111083	Gm48274	predicted gene, 48274 [Source:MGI Symbol;Acc:MGI:6097700]	no|up	yes|down	1
ENSMUSG0000112774	Gm36041	predicted gene, 36041 [Source:MGI Symbol;Acc:MGI:5595200]	yes|down	no|up	1
ENSMUSG0000020926	Adam11	a disintegrin and metallopeptidase domain 11 [Source:MGI Symbol;Acc:MGI:1098667]	yes|down	no|down	1
ENSMUSG0000036957	Lrfn3	leucine rich repeat and fibronectin type III domain containing 3 [Source:MGI Symbol;Acc:MGI:2442512]	yes|up	no|down	1
ENSMUSG0000052133	Sema5b	sema domain, seven thrombospondin repeats (type 1 and type 1-like), transmembrane domain (TM) and short cytoplasmic domain, (semaphorin) 5B [Source:MGI	yes|up	no|down	1

		Symbol;Acc:MGI:107555]			
ENSMUSG00000043488	Frmd8os	FERM domain containing 8, opposite strand [Source:MGI Symbol;Acc:MGI:3704490]	no|down	yes|up	1
ENSMUSG00000082016	Pgam1-ps2	phosphoglycerate mutase 1, pseudogene 2 [Source:MGI Symbol;Acc:MGI:3645709]	yes|down	no|no change	1

Table S4 Metabolome analysis of significant difference metabolites compared the HFD group with alliin group, according to the criteria of P<0.05 and FC≥1.5

Metabolite Name	Formula	Up or Down	Calc. MW	RT [min]	Area (Max.)
Cyprodenate	C13 H25 N O2	Up	227.18858	18.073	120517362.3
N-Undecanoylglycine	C13 H25 N O3	Up	243.18333	16.568	109696917.9
N-Desmethyltramadol	C15 H23 N O2	Up	249.17067	18.075	85548978.24
Myristyl sulfate	C14 H30 O4 S	Up	294.18681	20.643	56167058.48
Pivagabine	C9 H17 N O3	Up	187.12	8.008	27856378.39
(+/-)-Coniine	C8 H17 N	Up	127.13631	4.085	26468844.74
2-oxa-4-azatetracyclo[6.3.1.1~6,10~.0~1,5~]trid	C11 H15 N O2	Up	193.10793	8.813	22817042.38

ecan-3-one					
Aceclidine	C9 H15 N O2	Up	169.11039	8.018	15679393.96
Stearamide	C18H37NO	Up	283.28745	21.796	583208946.3
Morphine D3	C17 H16 [2]H3 N O3	Up	288.15497	6.812	14858362.96
D-pantothenic acid	C9 H17 N O5	Down	219.11057	3.786	177564458.8
Adipic acid	C6 H10 O4	Down	146.05689	2.837	93310379.43
2-amino-N-(1,3,5-trimethyl-1H-pyrazol-4-yl)benzamide	C13 H16 N4 O	Down	244.12893	6.569	79555990.15
2-Thio-acetyl MAGE	C21 H42 O3 S	Down	356.27139	21.207	26122535.15
Furaneol	C6 H8 O3	Down	128.04765	2.878	21199623.52
6β-Prostaglandin I1	C20 H34 O5	Down	354.24113	18.428	17407117.03
8-iso-13,14-dihydro-15-keto Prostaglandin F2?	C20 H34 O5	Down	354.24107	19.227	16637664.58
Amobarbital	C11H18N2O3	Down	226.13195	6.672	15224130.49

Table S4 exegesis: Calc. MW : Molecular Weight Calculator; RT: retention time; Area (Max.): Maximum peak area.

Table S5 There was significantly differential genes and metabolites enrichment pathway.

Pathway name	Total	Expected	Hits	p-value	-log(p)	Holm adjust	FDR	Impact
Steroid biosynthesis	19	0.12781	6	1.80E-09	8.7442	1.51E-07	1.51E-07	0.64103
Terpenoid backbone biosynthesis	23	0.15472	6	6.57E-09	8.1826	5.45E-07	2.76E-07	1.4706

Glycerolipid metabolism	61	0.41035	7	1.41E-07	6.8498	1.16E-05	3.96E-06	1.7222
Glycerophospholipid metabolism	97	0.65252	8	2.31E-07	6.6357	1.87E-05	4.86E-06	0.97959
Steroid hormone biosynthesis	89	0.5987	7	1.93E-06	5.7145	0.000154	3.24E-05	1.2887
Alanine, aspartate and glutamate metabolism	38	0.25563	4	0.00011476	3.9402	0.009066	0.001607	0.40625
Arginine biosynthesis	19	0.12781	3	0.00025934	3.5861	0.020228	0.003105	0.75
Drug metabolism - other enzymes	88	0.59197	5	0.00029574	3.5291	0.022772	0.003105	0.5
Tyrosine metabolism	40	0.26908	3	0.0023923	2.6212	0.18181	0.022328	0.91111
Retinol metabolism	91	0.61215	4	0.00319	2.4962	0.23925	0.026796	1.4118
Primary bile acid biosynthesis	16	0.10763	2	0.0050235	2.299	0.37174	0.038361	0.25581
Glycosaminoglycan biosynthesis - heparan sulfate / heparin	19	0.12781	2	0.0070665	2.1508	0.51586	0.045661	0
Fatty acid biosynthesis	19	0.12781	2	0.0070665	2.1508	0.51586	0.045661	0.066667
Glutathione metabolism	65	0.43725	3	0.0093942	2.0271	0.66699	0.056365	0.7037
Phenylalanine metabolism	23	0.15472	2	0.010277	1.9881	0.71937	0.05755	0.90909
Glyoxylate and dicarboxylate metabolism	31	0.20854	2	0.018251	1.7387	1	0.095816	0.47826
Glycine, serine and threonine metabolism	40	0.26908	2	0.029459	1.5308	1	0.14556	1.2895
Neomycin, kanamycin and gentamicin	5	0.033635	1	0.033193	1.479	1	0.1549	0

biosynthesis								
Ether lipid metabolism	47	0.31617	2	0.039629	1.402	1	0.16674	0.33333
Phosphonate and phosphinate metabolism	6	0.040362	1	0.0397	1.4012	1	0.16674	0.33333
Fatty acid degradation	50	0.33635	2	0.044339	1.3532	1	0.1693	0.048387
Arginine and proline metabolism	50	0.33635	2	0.044339	1.3532	1	0.1693	0.20513
Cysteine and methionine metabolism	52	0.3498	2	0.04759	1.3225	1	0.17381	0.27027
Phenylalanine, tyrosine and tryptophan biosynthesis	8	0.053816	1	0.052587	1.2791	1	0.18405	0.33333
Pyrimidine metabolism	58	0.39016	2	0.057838	1.2378	1	0.19434	0.32203
Taurine and hypotaurine metabolism	11	0.073997	1	0.071598	1.1451	1	0.23132	0.57143
Metabolism of xenobiotics by cytochrome P450	67	0.45071	2	0.074482	1.1279	1	0.23172	0.62295
Drug metabolism - cytochrome P450	69	0.46416	2	0.078368	1.1059	1	0.2351	0.11765
Arachidonic acid metabolism	89	0.5987	2	0.12032	0.91965	1	0.34852	2.2619
Various types of N-glycan biosynthesis	20	0.13454	1	0.12641	0.89822	1	0.35395	0.27586
alpha-Linolenic acid metabolism	25	0.16817	1	0.15547	0.80834	1	0.42128	0.25
Ascorbate and aldarate metabolism	27	0.18163	1	0.16683	0.77772	1	0.43793	0.33333
Mucin type	28	0.1883	1	0.17245	0.763	1	0.4389	0.4444

O-glycan biosynthesis		6			33		7	4
Pentose phosphate pathway	32	0.21526	1	0.19457	0.71092	1	0.45398	0
Galactose metabolism	32	0.21526	1	0.19457	0.71092	1	0.45398	0.13043
Propanoate metabolism	33	0.22199	1	0.20001	0.69895	1	0.45398	0.083333
Starch and sucrose metabolism	33	0.22199	1	0.20001	0.69895	1	0.45398	0.095238
Pentose and glucuronate interconversions	34	0.22872	1	0.20541	0.68737	1	0.45398	0.15385
Fructose and mannose metabolism	35	0.23544	1	0.21078	0.67617	1	0.45398	0.22222
Pyruvate metabolism	38	0.25563	1	0.22666	0.64462	1	0.476	0.18182
Porphyrin and chlorophyll metabolism	41	0.27581	1	0.24224	0.61576	1	0.49629	0.090909
Tryptophan metabolism	48	0.32289	1	0.27738	0.55692	1	0.52432	0.52381
Sphingolipid metabolism	48	0.32289	1	0.27738	0.55692	1	0.52432	0.63889
Amino sugar and nucleotide sugar metabolism	49	0.32962	1	0.28227	0.54933	1	0.52432	0.068182
N-Glycan biosynthesis	50	0.33635	1	0.28713	0.54193	1	0.52432	0.052632
Linoleic acid metabolism	50	0.33635	1	0.28713	0.54193	1	0.52432	1.0909
Valine, leucine and isoleucine degradation	56	0.37671	1	0.31559	0.50088	1	0.56403	0.081633
Aminoacyl-tRNA biosynthesis	66	0.44398	1	0.36057	0.44301	1	0.61812	0
Glycolysis or Gluconeogenesis	66	0.44398	1	0.36057	0.44301	1	0.61812	0.20588

Phosphatidylino sitol signaling system	98	0.6592 4	1	0.48585	0.313 5	1	0.8162 2	0.0833 33
Purine metabolism	136	0.9148 7	1	0.60357	0.219 27	1	0.9941 1	0.1078 4

Table S5 exegesis: The overview shows all matched pathways according to the p-value from the pathway enrichment analysis and pathway impact values from the pathway topology analysis.
